# Predictors of Long-COVID and Chronic Impairment of Exercise Tolerance in Spiroergometry in Patients after 15 Months of COVID-19 Recovery

**DOI:** 10.3390/jcm12247689

**Published:** 2023-12-14

**Authors:** Katarzyna Gryglewska-Wawrzak, Agata Sakowicz, Maciej Banach, Agata Bielecka-Dabrowa

**Affiliations:** 1 Department of Cardiology and Congenital Diseases of Adults, Polish Mother’s Memorial Hospital Research Institute (PMMHRI), 93-338 Lodz, Poland; maciejbanach77@gmail.com (M.B.); agatbiel7@poczta.onet.pl (A.B.-D.); 2 Department of Medical Biotechnology, Medical University of Lodz, 90-131 Lodz, Poland; agata.sakowicz@gmail.com; 3 Department of Preventive Cardiology and Lipidology, Medical University of Lodz, 90-131 Lodz, Poland

**Keywords:** COVID-19, long COVID, spiroergometry, maximal oxygen uptake, body mass analysis

## Abstract

Background: The aim of the study was to identify factors that may cause the presence of long COVID and to assess factors that affect chronic limited exercise tolerance in spiroergometry after one-year follow-up in patients who had recovered from COVID-19. Methods: Of 146 patients hospitalised in the Cardiology Department, 82 completed a one-year follow-up (at least 15 months post-COVID-19 recovery). We compared their conditions at initial screening and follow-up to analyse the course of long COVID and exercise intolerance mechanisms. Clinical examinations, laboratory tests, echocardiography, cardiopulmonary exercise testing, and body composition analysis were performed. Results: The patients, after one-year follow-up, had significantly higher levels of high-sensitivity cardiac troponin T (hs-cTnT) (*p* = 0.03), left atrium diameter (LA) (*p* = 0.03), respiratory exchange ratio (RER) (*p* = 0.008), and total body water content percentage (TBW%) (*p* < 0.0001) compared to the 3-month assessment. They also had lower forced vital capacity in litres (FVC) (*p* = 0.02) and percentage (FVC%) (*p* = 0.001). The factors independently associated with a decline in maximum oxygen uptake (VO_2max_) after one-year follow-up included the percentage of fat (OR 2.16, 95% CI: 0.51–0.77; *p* = 0.03), end-diastolic volume (EDV) (OR 2.38, 95% CI 0.53–0.78; *p* = 0.02), and end-systolic volume (ESV) (OR 2.3, 95% CI: 0.52–0.78; *p* = 0.02). Conclusions: Higher left ventricular volumes and fat content (%) were associated with a reduced peak VO_2max_ when assessed 15 months after COVID-19 recovery.

## 1. Introduction

COVID-19 is a viral respiratory illness caused by severe acute respiratory syndrome coronavirus 2 (SARS-CoV-2). The outbreak began as an epidemic on 17 November 2019, in Wuhan, located in central China, and was officially classified as a pandemic by the World Health Organisation (WHO) on 11 March 2020. The COVID-19 pandemic caused an unprecedented disturbance in healthcare worldwide [1,2,3]. Although many individuals recover fully from COVID-19, a significant number continue to experience persistent symptoms and functional limitations, even after a year or more from the initial diagnosis [4,5]. One of the most common symptoms reported in these individuals is exercise intolerance, which can greatly impact their quality of life. Understanding the predictors of chronic impairment of exercise tolerance in patients after COVID-19 is crucial for better management and rehabilitation strategies [6]. Furthermore, it should be noted that the long-term effects of COVID-19 are still actively being researched. New information about the virus and its impact on different body systems continues to emerge. Therefore, the list of predictors can evolve as researchers gain a deeper understanding of the long-term consequences of the disease. Assessing exercise tolerance using spiroergometry can provide valuable insight into the functional capacity of individuals after COVID-19 [7]. It allows the measurement of oxygen consumption, carbon dioxide production, and other respiratory parameters during exercise [8]. By identifying the predictors of chronic impairment of exercise tolerance, healthcare professionals can develop customised rehabilitation programmes that address specific limitations and improve overall fitness and well-being in patients after COVID-19. It is important to emphasise the multidisciplinary approach to the management of patients post-COVID-19, including respiratory physicians, cardiologists, physiotherapists, and other relevant specialists [9]. Comprehensive evaluations, including spiroergometry, along with clinical evaluations and medical imaging, can help identify predictors and guide the development of appropriate treatment strategies to optimise recovery and enhance exercise tolerance in patients after COVID-19 [10]. This study aimed to investigate the underlying mechanisms of these symptoms in patients with long-COVID. The purpose of the study was to compare and identify factors that contribute to chronic limited exercise tolerance in patients after one year of follow-up after COVID-19.

## 2. Materials and Methods

### 2.1. Basic Characteristics

Out of the 146 consecutive patients (38% males, 62% females) who had been hospitalised in the Department of Cardiology and had recovered from COVID-19 3 to 6 months after confirmed diagnosis, 82 (56%) (35 males, 47 females) completed a 1-year follow-up, which occurred at least 15 months after the initial COVID-19 diagnosis. Patients were assigned to appropriate groups based on the WHO definition of long COVID. The long COVID patients presented with the continuation or development of new symptoms 3 months after the initial SARS-CoV-2 infection, with these symptoms lasting for at least 2 months with no other explanation [11]. We compared the same patients’ condition after 3–6 months and after 1-year follow-up from recovery to establish the course of long-COVID and the mechanism of chronic exercise intolerance after COVID-19. The average age of patients at inclusion was 54. Every participant included in the study conducted a cardiopulmonary exercise testing (CPET) on the ergometer. The patients did not participate in rehabilitation programs and did not increase their daily physical activities during the interval between assessments. The patients in the study groups maintained a sedentary lifestyle. The study was approved by the Polish Mother’s Memorial Hospital Research Institute (PMMHRI-BCO.75/2020) and is in compliance with the Declaration of Helsinki [7].

Exclusion criteria:

- Heart failure diagnosis or typical symptomatic heart failure;
- Previous myocardial infarction;
- Uncontrolled arterial hypertension;
- Unstable angina;
- Acute pericarditis or myocarditis;
- Active endocarditis;
- Acute pulmonary embolism;
- History of stroke, transient ischemic attack, or intracerebral haemorrhage;
- Unstable heart rhythm disorders;
- Advanced atrioventricular block;
- Cardiomyopathy diagnosis (dilated, hypertrophic, postpartum, restrictive, tachyarrhythmic);
- Active systemic infection;
- Carrier of Hepatitis B virus (HBV), Hepatitis C virus (HCV), or human immunodeficiency virus (HIV), or testing positive for hepatitis B surface antigen (HBsAg) or antibodies to HCV;
- Drug and alcohol abuse;
- Chronic kidney disease (stage IV and V according to the National Kidney Foundation) and dialysis treatment;
- Severe hypothyroidism and hyperthyroidism;
- Active autoimmune disease;
- Use of cytostatic drugs, immunosuppressants, glucocorticosteroids, or antiretroviral drugs;
- Documented neoplastic process;
- History of bone marrow transplantation or other organ transplantation, as well as treatment with blood products within the last 6 months;
- Underwent surgery or a significant injury in the last month;
- Pregnancy or lactation;
- A physical limitation that hinders the completion of a spiroergometric test;
- Patient’s incapacity to cooperate and/or provide informed consent to participate in research;
- Individuals who did not provide their informed consent to take part in the study [7].

### 2.2. Echocardiography

Echocardiograms were conducted using the Vivid E95 system (GE Healthcare, Chicago, IL, USA) [7]. Measurements were performed according to current guidelines [12]. To determine the volume and ejection fraction (EF) of the left ventricular (LV), the modified Simpson’s rule was employed. The estimation of the left atrial (LA) volume utilised the modified biplane Simpson’s method, employing apical 2- and 4-chamber views at end-systole. The resulting volume was indexed to the patient’s body surface area to derive the LA volume index (LAVi) [13]. Various other measurements were analysed, including maximal early (E) and late (A) transmitral velocities, as well as the ratio of early to late diastolic transmitral flow velocity (E/A) [14]. Global peak systolic strain (GLPS) assessment was conducted via speckle tracking echocardiography [15]. Evaluation of right ventricular (RV) function involved obtaining measurements such as tricuspid annular plane systolic excursion (TAPSE) and tissue Doppler echocardiography (TDE) [16].

### 2.3. Spiroergometry

Cardiopulmonary exercise testing (CPET) was performed using an upright cycle ergometer called Bike M, which features electromagnetic braking (CORTEX Biophysik GmbH, Leipzig, Germany) [7]. Metabolic gas analysis was performed using the METALYZER 3B system (CORTEX Biophysik GmbH, Leipzig, Germany) with the assistance of the MetaSoft Studio application software (version 5.8.3) developed by CORTEX systems [17]. Before each test, the system underwent calibration using a standard gas mixture of known concentrations. The breath gas analyser was internally calibrated just before each measurement. Both the volume of gases and the flow sensor were calibrated immediately before the test, with their calibration validated twice yearly. Following a 5-min rest on the cycle ergometer, exercise began at 50 W, and, subsequently, the workload was raised by 25 W every 3 min. Before exercise testing on the bicycle ergometer, spirometry was conducted to assess lung function. Forced expiratory volume in one second (FEV_1_) and forced vital capacity (FVC) were assessed. The FEV_1_/FVC ratio, also known as the Tiffeneau index, was recorded [18]. During CPET on the bicycle ergometer, the continuous monitoring of the non-invasive blood pressure (NIBP) using a sphygmomanometer Exacta (Rudolf Riester GmbH, Jungingen, Germany), heart rate (HR), 12-lead electrocardiogram (ECG) using an exercise electrocardiogram Meta control 3000 (CORTEX Biophysik GmbH, Leipzig, Germany), and oxygen saturation (SpO_2_) were carried out. One of the key measurements obtained during CPET is maximum oxygen uptake (VO_2max_), which represents the highest level of oxidative metabolism achievable by engaging large muscle groups. If a clear plateau is not observed in the oxygen uptake curve, the highest VO_2_ achieved, referred to as VO_2_ peak, can be used as a substitute for VO_2max_ [19]. Furthermore, several other valuable CPET parameters were evaluated. These derived measurements include ventilatory exchange (VE), oxygen uptake (VO_2_), carbon dioxide expenditure (VCO_2_), respiratory exchange ratio (RER), anaerobic threshold (AT), oxygen uptake at the anaerobic threshold (VO_2_AT), and the slope of minute ventilation to carbon dioxide production (VE/VCO_2_ slope) [20].

### 2.4. Body Mass Analysis

The Segmental Body Composition Analyser (Tanita Pro, Tokyo, Japan) is a non-invasive tool used for analysing body mass [7]. This device utilises various methods, such as Dual-energy X-ray absorptiometry (DXA) and the dilution method, for total body water measurement employing the Bioelectrical Impedance Analysis (BIA) method [21]. The analyser then proceeded to provide separate mass readings for different body segments and estimated values for total, regional fat mass (FM), and fat-free mass (FFM). Furthermore, measurements were taken for total body water (TBW), intracellular water (ICW), and extracellular water (ECW). The correlation involving ECW/TBW, defined as the ECW/TBW percentage ratio, and basal metabolic rate (BMR) were also examined [22].

### 2.5. Statistical Analysis

Analysis was performed using the STATISTICA 13.1 software package (StatSoft, Krakow, Poland). Normal distribution was assessed using the Shapiro–Wilk test. The Wilcoxon signed-rank test was applied for variables. Significant continuous data from univariate analyses were used to create receiver-operating characteristic (ROC) curves, and the Youden index was utilised to convert these into categorical data. Backward stepwise multivariate logistic regression tested these categorical data. Results were deemed significant at a threshold of *p* < 0.05.

## 3. Results

### 3.1. Evaluation of Basic Characteristics

We compared 82 consecutive patients, who were hospitalised in the Department of Cardiology and had recovered from COVID-19 three to six months after confirmed diagnosis, examining the same patients during a one-year follow-up. There were no differences in medical treatment between the groups. The data are presented in Table 1.

### 3.2. Evaluation of Laboratory Tests

Follow-up subjects presented a significantly higher level of hs-cTnT (median 5.8 (IQR: 3.9–9.3) vs. 4.9 (IQR: 3.2–8.4) pg/mL, *p* = 0.03). There were no statistically significant differences in residual laboratory parameters. The data are shown in Table 2.

### 3.3. Evaluation of Echocardiography

The LA diameter was significantly higher in patients in one year follow-up [median 38 (IQR: 34–43) vs. 36 (IQR: 34–43) mm, *p* = 0.03]. The results are presented in Table 3.

### 3.4. Evaluation of Spiroergometry

During follow-up, the subjects presented with higher RER (median 1.10 (IQR: 1.05–1.13) vs. 1.09 (IQR: 1.02–1.12), *p* = 0.008) and lower FVC (L), FVC (%) (median 3.71 (IQR: 2.88–4.27) vs. 3.79 (IQR: 3.18–4.44) L, *p* = 0.02; median 101 (IQR: 91–112) vs. 105 (IQR: 95–117) %, *p* = 0.001; respectively). There were no significant differences with respect to the other parameters. The data are demonstrated in Table 4.

### 3.5. Evaluation of Body Mass Analysis 

Only TBW content in % was statistically significantly elevated in follow-up patients (median 53.1 (IQR: 49.3–57.9) vs. 49.5 (IQR: 47.8–56.0)%, *p* < 0.0001). The results are shown in Table 5.

### 3.6. Multivariate Analysis 

**Figure 1:**
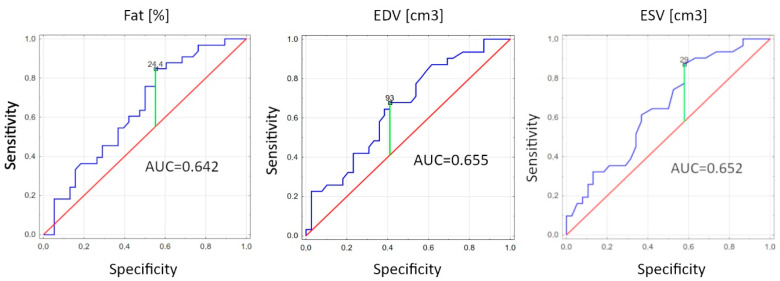
Receiver-operating curve (ROC) for the fat (%), end-diastolic volume (EDV) and end-systolic volume (ESV) variables revealing their diagnostic potential. AUC—area under the ROC curve; blue line—graphical plot of studied parameter determining the area under the curve, green line—the line pointed at Youden point, red line—reference line.

In a multiple logistic regression model, the two factors were found to be significantly associated with a worsening of VO_2max_ in the follow-up: percentage of fat (OR 2.16, 95% CI: 0.51–0.77; *p* = 0.03), end-diastolic volume (EDV) (OR 2.38, 95% CI 0.53–0.78; *p* = 0.02), and end-systolic volume (ESV) (OR 2.3, 95% CI: 0.52–0.78; *p* = 0.02). The results are presented in Table 6 and Figure 1.

## 4. Discussion

The findings of this study suggest that several factors contribute to the chronic impairment of exercise tolerance in patients after COVID-19. At one year of follow-up, pateints presented elevated levels of hs-cTnT. In a prior study, where we compared patients three to six months after their COVID-19 diagnosis, subjects with symptoms also exhibited higher levels of hs-cTnT [7]. It is important to underscore that hs-cTnT remained within the boundaries of the normal range in both groups under comparison. The findings emphasise that the differences observed between these two groups do not signify an acute cardiac issue or significant myocardial damage, at least as assessed by hs-cTnT levels. Hs-cTnT is a specific biomarker used in the diagnosis and evaluation of heart-related conditions [23]. The Hs-cTnT test utilises advanced techniques that can detect lower levels of troponin T in the blood with increased sensitivity and precision [24]. This allows the early detection of myocardial injury and a more accurate assessment of cardiac conditions. The test measures the concentration of hs-cTnT in the blood and compares it with established reference ranges to aid in the diagnosis and stratification of patients with suspected heart-related problems. The clinical significance of troponin elevation, even when still within the normal range, and its impact on cardiovascular outcomes are still uncertain. In contrast to our results, there are few studies on persistently elevated troponin levels in post-COVID-19 patients. In a study by Kotecha et al., cardiac magnetic resonance imaging (CMR) revealed late gadolinium and/or ischaemia in 54% of patients with severe COVID-19 and persistent elevation of troponin elevation at around 68 days after discharge [25]. Similar CMR findings have been observed in patients with mild COVID-19 six months after infection compared to healthy individuals, as demonstrated by Joy et al. [26]. However, the long-term implications of these findings and their clinical relevance are yet to be determined. 

Our next findings indicated that follow-up subjects had a higher RER. Furthermore, in the aforementioned previous research study, patients without symptoms three to six months after a COVID-19 diagnosis also exhibited higher RER [7]. The RER is a physiological parameter that indicates the ratio between carbon dioxide (CO_2_) production and oxygen (O_2_) consumption during cellular respiration. It is calculated by dividing the volume of carbon dioxide produced (VCO_2_) by the volume of oxygen consumed (VO_2_). The RER value provides insights into whether the body is primarily using carbohydrates (RER close to 1.0) or fats (RER close to 0.7) for energy [27,28]. An RER exceeding 1.0 indicates an increasing reliance on anaerobic metabolism, which often accompanies higher exercise intensity and a transition to less sustainable energy sources. Notably, an RER surpassing 1.10 is commonly used as a criterion for exhaustion [29]. In the study of Joris et al., CPET was performed in critically ill survivors [30]. Their metabolic efficiency was low at 15.2 (12.9–17.8)%. The 50% decrease in VO_2_ after maximum effort was delayed, at 130 (120–170) s, with a RER that was still high (1.13 [1–1.2]). In the presented study, the higher median RER in the follow-up group (1.10) compared to the comparison group (1.09) suggests an increased reliance on carbohydrates as an energy source in the follow-up patients. The elevated RER in the follow-up group might suggest altered metabolic responses or increased reliance on anaerobic metabolism during exercise. The significance of these results could prompt further investigations into the underlying mechanisms contributing to the observed differences in RER.

Total body water (TBW) refers to the total amount of water present in a person’s body. It represents the sum of water content in all body compartments, including intracellular and extracellular fluid [31]. TBW plays a vital role in various physiological processes, such as maintaining hydration, regulating body temperature, transporting nutrients, facilitating waste removal, and supporting cellular function. The exact TBW value can vary depending on factors such as age, sex, body composition, and overall health. On average, TBW accounts for about 50–70% of the total body weight in adults, with higher percentages typically seen in infants and children due to their higher water content relative to body weight [32]. The TBW content can be estimated using the BIA method. In our study, we demonstrated that the follow-up patients had higher TBW content. In our other study, we showed that hydration status is related to limited exercise tolerance after 3 to 6 months after the diagnosis of COVID-19 in patients with normal LVEF [6]. However, in the comparison of patients three to six months after their COVID-19 diagnosis, there were no statistically significant differences regarding hydration status [7]. Some studies investigated the association between body mass compartments and the severity of COVID-19. Cornejo-Pareja demonstrated that overhydration was an important predictor of COVID-19 mortality [33].

Forced vital capacity (FVC) is a commonly used measurement in pulmonary function testing to assess lung function. It is a measure of the maximum amount of air that a person can forcefully exhale after taking a deep breath. FVC is an essential parameter in the diagnosis and monitoring of various respiratory diseases, such as chronic obstructive pulmonary disease (COPD), asthma, and restrictive lung diseases [34]. It is important to note that FVC measurements are influenced by several factors, including age, sex, body position during testing, patient effort, and the presence of any respiratory muscle weakness or abnormalities of the respiratory muscles [35]. In this study, the follow-up subjects presented with decreased FVC. Furthermore, in the previously mentioned study, patients with symptoms presented a decreased FVC [7]. Some clinical observations have indicated that long-COVID can lead to persistent respiratory symptoms and abnormalities in lung function, including reduced FVC [36,37,38].

Our findings revealed that left ventricular volumes and fat content are associated with a reduced VO_2max_ 15 months after COVID-19 recovery. In our previous study, there was no statistical significance regarding these parameters [7]. Brown et al. conducted a study on exercise, examining discharged patients with COVID-19 who self-reported reduced exercise capacity. They compared them to discharged patients with normal exercise capacity, as well as a control group [39]. When adjusted for body surface area, the individuals with a history of COVID-19 exhibited a decrease in left ventricular end-systolic volume indexed to body surface area and an increase in left ventricular ejection fraction. Furthermore, those with reduced exercise capacity showed reduced index oxygen consumption, indexed stroke volume, and indexed left ventricular end-diastolic volume. In another study, 346 people with prior COVID infection underwent a baseline examination after a minimum of 4 weeks from the initial diagnosis of COVID-19 between April 2020 and October 2021 and a follow-up examination after a minimum of 4 months from baseline. The authors showed that female sex and small LV volumes and masses were associated with symptomatic status at follow-up [40]. The fat content in the body, specifically in terms of body composition, plays a significant role in overall health. Excess body fat, particularly when it accumulates in excess, can have implications for various aspects of health [41]. Mondal et al. conducted a study in which they presented findings that increased body fat is associated with a decreased level of VO_2_ peak [42]. However, in another study, the authors revealed that the main influence of body weight on VO_2max_ is explained by FFM, and fat content does not have any effect on oxygen consumption [43].

Furthermore, it is noteworthy that the patients in this study led sedentary lifestyles and did not participate in rehabilitation programs. This sedentary lifestyle could have contributed to the observed physiological changes, as physical inactivity can lead to deconditioning and further exacerbate exercise intolerance in long COVID patients. Incorporating rehabilitation programs tailored to the specific challenges faced by long COVID patients may play a significant role in improving their exercise tolerance and overall health.

## 5. Limitations

The presented study has certain limitations that should be taken into consideration. First, the study population consisted of a relatively small sample size, with only 82 participants. Additionally, the study design did not thoroughly evaluate the potential effects of the medications used by the participants. Furthermore, the study involved only patients capable of performing CPET. There were also limitations in terms of the measurements conducted. Diffusion lung capacity for carbon monoxide (DLCO) and total lung capacity (TLC) were not measured, which could provide valuable insights into lung function and gas exchange. Additionally, TTE was performed only at rest, and certain echocardiographic parameters, such as left atrial strain, were not obtained. Given these limitations, caution should be exercised when interpreting the data from this study. It is recommended that future studies address these limitations by including larger post-COVID populations and incorporating measurements of TLC and DLCO. Furthermore, conducting TTE assessments during exercise would further enhance our understanding of cardiac implications in post-COVID patients.

The strengths of our study lie in its pioneering approach as one of the first investigations to explore the potential of selected echocardiographic, laboratory, and spiroergometric parameters in the evaluation of patients after COVID-19 during a one-year follow-up.

## 6. Conclusions

In conclusion, after a 15 months period after COVID-19 recovery, patients showed elevated levels of hs-cTnT, RER, TBW%, and reduced FVC. Higher left ventricular volumes and fat content (%) were associated with a reduced peak VO_2max_ assessed 15 months after COVID-19 recovery. These findings shed light on the factors that contribute to chronic exercise intolerance in patients after COVID-19 and emphasise the importance of the long-term monitoring and treatment of individuals affected by long COVID.

## Figures and Tables

**Table 1:** Evaluation of basic characteristics.

Parameter	Patients Hospitalised Three to Six Months after COVID-19 Diagnosis *n* = 82	Patients Hospitalised in One-Year Follow-Up *n* = 82	*p*
Clinical characteristics			
BMI (kg/m^2^)	(23.24–30.42), 26.79 *	(24.03–30.25), 26.28 *	0.32
BSA (m^2^)	(1.75–2.03), 1.86 *	(1.76–2.05), 1.88 *	0.23
SBP (mmHg)	(125.00–140.00), 130.00 *	(125.00–145.00), 132.00 *	0.10
DBP (mmHg)	(71.00–86.00), 80.00 *	(75.00–90.00), 82.00 *	0.052
Hypertension	57%	61%	0.63
Diabetes mellitus	20%	21%	0.85
Dyslipidaemia	54%	57%	0.69
Obesity	24%	29%	0.48
Alcohol	4%	2%	0.68
Smoking	11%	16%	0.36

*—median; values with non-normal distribution are expressed as median (range) values. Values with normal distributions are expressed as mean ± standard deviation (SD). BMI—body mass index; BSA—body surface area; SBP—systolic blood pressure; DBP—diastolic blood pressure.

**Table 2:** Evaluation of laboratory tests among investigated groups.

Parameter	Patients Hospitalised Three to Six Months after COVID-19 Diagnosis *n* = 82	Patients Hospitalised in One-Year Follow-Up *n* = 82	*p*
Laboratory tests			
hs-cTnT (pg/mL) (<14.00)	(3.20–8.40), 4.90 *	(3.90–9.30), 5.80 *	0.03
NT-proBNP (pg/mL) (<125.00)	(42.00–125.00), 87.00 *	(51.00–120.00), 82.00 *	0.80
RBC (10^6^/μL) (women—3.80–5.80, men—4.50–6.50)	(4.20–4.90), 4.50 *	(4.20–4.90), 4.50 *	0.37
Haemoglobin (g/dL) (women—12.00–15.00; men—13.00–18.00)	(12.80–14.60), 13.70 *	(12.60–14.80), 13.40 *	0.32
PLT (10^3^/μL) (150.00–400.00)	(165.00–224.00), 205.00 *	(172.00–238.00), 210.00 *	0.70
Creatinine (mg/dL) (0.55–1.02)	(0.67–0.91), 0.79 *	(0.65–0.93), 0.77 *	0.97
GFR (ml/min/1.73 m^2^) (>90.00)	(79.10–104.10), 92.20 *	(79.60–103.80), 91.50 *	0.26
Urea (mg/dL) (17.00–43.00)	(27.00–40.00), 32.00 *	(27.00–38.00), 41.00 *	0.59
Glucose (mg/dL) (60.00–99.00)	(85.00–96.00), 90.00 *	(84.00–96.00), 91.00 *	0.66
HDL cholesterol (mg/dL) (>40.00)	(39.00–59.00), 49.50 *	(40.00–57.00), 47.50 *	0.58
LDL cholesterol (mg/dL) (<115.00)	(70.00–110.00), 94.00 *	(62.00–106.00), 85.00 *	0.27
Triglycerides (mg/dL) (<150.00)	(83.00–148.00), 103.50 *	(78.00–130.00), 100.00 *	0.07
Total cholesterol (mg/dL) (<200.00)	(135.00–188.00), 169.00 *	(130.00–188.00), 161.00 *	0.33
ALT (U/L) (<50.00)	(17.00–29.00), 22.00 *	(17.00–30.00), 22.00 *	0.59
AST (U/L) (<50.00)	(25.00–31.00), 27.00 *	(25.00–32.00), 28.00 *	0.73
CRP (mg/dL) (<0.50)	(0.50–0.50), 0.50 *	(0.50–0.50), 0.50 *	0.22
D-dimer (ng/mL) (<500.00)	(200.50–398.50), 286.00 *	(167.00–326.00), 224.00 *	0.19
K (mmol/L) (3.50–5.10)	(4.20–4.60), 4.40 *	(4.20–4.60), 4.40 *	0.78
Na (mmol/L) (135.00–145.00)	(138.00–141.00), 139.00 *	(138.00–141.00), 140.00 *	0.29

*—median; values with non-normal distribution are expressed as median (range) values. Values with normal distributions are expressed as mean ± standard deviation (SD). Hs-cTnT—high-sensitivity cardiac troponin; NT-proBNP—N-terminal prohormone of brain natriuretic peptide; RBC—red blood cells; PLT—thrombocytes; GFR—glomerular filtration rate; HDL—high-density lipoprotein; LDL—low-density lipoprotein; ALT—alanine aminotransferase; AST—aspartate aminotransferase; CRP—c-reactive protein; K—serum potassium; Na—serum sodium.

**Table 3:** Evaluation of selected echocardiographic parameters among investigated groups.

Parameter	Patients Hospitalised Three to Six Months after COVID-19 Diagnosis *n* = 82	Patients Hospitalised in One-Year Follow-Up *n* = 82	*p*
Echocardiography			
EF (%)	(55.00–65.00), 62.00 *	(58.00–65.00), 63.00 *	0.27
EDV (cm^3^)	(75.00–105.00), 92.00 *	(77.00–117.00), 99.00 *	0.055
ESV (cm^3^)	(26.00–48.00), 37.00 *	(28.00–48.00), 37.00 *	0.26
LA (mm)	(34.00–43.00), 36.00 *	(34.00–43.00), 38.00 *	0.03
LAVi (ml/m^2^)	(26.00–41.00), 34.00 *	(27.00–42.00), 33.00 *	0.45
E (cm/s)	(62.00–87.00), 75.00 *	(63.00–96.00), 78.00 *	0.43
A (cm/s)	(54.00–80.00), 67.00 *	(54.00–75.00), 65.00 *	0.85
E/A	(0.87–1.44), 1.12 *	(0.92–1.49), 1.14 *	0.25
GLPS (%)	(18.80–20.80), 20.00 *	(18.10–20.10), 19.10 *	0.94
TAPSE (mm)	(20.00–26.00), 23.00 *	(20.00–25.00), 22.00 *	0.63
TDE S’ (cm/s)	(12.00–15.00), 13.00 *	(11.00–15.00), 13.00 *	0.06

*—median; values with non-normal distribution are expressed as median (range) values. Values with normal distributions are expressed as mean ± standard deviation (SD). EF—left ventricular ejection fraction; EDV—end-diastolic volume; ESV—end-systolic volume; LA—left atrium; LAVi—left atrial volume index; E—early diastolic filling velocity, A—late diastolic filling velocity; E/A—ratio of early to late diastolic transmitral flow velocity; GLPS—global peak systolic strain; TAPSE—tricuspid annular plane systolic excursion; TDE S’—tissue Doppler echocardiography.

**Table 4:** Evaluation of spiroergometry among investigated groups.

Parameter	Patients Hospitalised Three to Six Months after COVID-19 Diagnosis *n* = 82	Patients Hospitalised in One-Year Follow-Up *n* = 82	*p*
Spiroergometry			
Exercise time (s)	(402.00–696.00), 518.00 *	(420.00–756.00), 609.00 *	0.30
HR max	(122.00–164.00), 146.00 *	(124.00–166.00), 142.00 *	0.59
Peripheral SBP max (mmHg)	(140.00–200.00), 160.00 *	(150.00–190.00), 165.00 *	0.63
Peripheral DBP max (mmHg)	(70.00–90.00), 80 *	(80.00–90.00), 80.00 *	0.02
FEV_1_ (l)	(2.55–3.56), 2.99 *	(2.45–3.53), 3.04 *	0.07
FVC (l)	(3.18–4.44), 3.79 *	(2.88–4.27), 3.71 *	0.02
FVC%	(95.00–117.00), 105.00 *	(91.00–112.00), 101.00 *	0.001
FEV_1_/FVC	(76.00–85.00), 82.00 *	(77.00–88.00), 82.00 *	0.16
FEV_1_/FVC%	(96.00–109.00), 103.00 *	(97.00–111.00), 103.00 *	0.16
FEF 25–75 (l/s)	(1.88–3.35), 2.72 *	(2.04–3.74), 3.02 *	0.34
RER	(1.02–1.12), 1.09 *	(1.05–1.13), 1.10 *	0.008
VO_2max_ (ml/min/kg)	(17.00–26.00), 21.00 *	(18.00–26.00), 22.00 *	0.12
VO_2max pred_ (%)	(71.00–104.00), 81.00 *	(71.00–98.00), 85.00 *	0.53
VO_2_AT (ml/min/kg)	(11.00–18.00), 14.00 *	(11.00–16.00), 13.00 *	0.38
Peak VO_2max_ (l)	(1.25–1.98), 1.65 *	(1.32–1.98), 1.63 *	0.56
VE/VCO_2_ slope	(25.60–32.70), 29.60 *	(26.50–33.30), 29.10 *	0.67

*—median; values with non-normal distribution are expressed as median (range) values. Values with normal distributions are expressed as mean ± standard deviation (SD). DBP—diastolic blood pressure; SBP—systolic blood pressure; FEV_1_—forced expiratory volume in one second; FVC—forced vital capacity; FEV_1_/FVC—ratio of forced expiratory volume in one second to forced vital capacity; FEF 25–75%—forced expiratory flow over the middle one half of the FVC; RER—respiratory exchange ratio; VO_2max_—the maximum amount of oxygen the body can utilise during a specified period of usually intense exercise; VO_2max pred_—predicted value of VO_2max_; VO_2_AT—oxygen uptake at anaerobic threshold per kilogram; peak VO_2_—highest respiratory oxygen uptake (VO_2_) achieved by the subject during the maximal exercise; VE/VCO_2_ slope—the minute ventilation/carbon dioxide production slope.

**Table 5:** Evaluation of body mass analysis among investigated groups.

Parameter	Patients Hospitalised Three to Six Months after COVID-19 Diagnosis *n* = 82	Patients Hospitalised in One-Year Follow-Up *n* = 82	*p*
Body mass analysis			
Fat (%)	(22.30–34.10), 29.20 *	(23.80–33.40), 27.90 *	0.96
Fat (kg)	(16.00–29.20), 23.60 *	(17.70–28.90), 21.60 *	0.45
FFM (kg)	(48.00–62.50), 55.50 *	(48.80–64.50), 55.70 *	0.67
TBW (kg)	(34.30–44.70), 39.50 *	(35.10–48.10), 41.80 *	0.07
TBW (%)	(47.80–56.00), 49.50 *	(49.30–57.90), 53.10 *	<0.0001
ECW (kg)	(15.30–19.60), 17.20 *	(15.20–20.90), 17.70 *	0.06
ICW (kg)	(19.40–25.50), 22.40 *	(20.50–27.30), 24.10 *	0.17
ECW/TBW × 100%	(40.80–45.30), 43.30 *	(40.70–45.50), 43.40 *	0.93

*—median; values with non-normal distribution are expressed as median (range) values. Values with normal distributions are expressed as mean ± standard deviation (SD). FFM—fat-free body mass; TBW—total body water; ECW—extracellular water; ICW—intracellular water, ECW/TBW%—ratio of extracellular water to total body water

**Table 6:** Multivariate analysis.

Variable	OR	95% CI for OR		*p*
		Lower Limit	Upper Limit	
Fat (%)	2.16	0.51	0.77	0.03
EDV (cm^3^)	2.38	0.53	0.78	0.02
ESV (cm^3^)	2.30	0.52	0.78	0.02

EDV—end-diastolic volume; ESV—end-systolic volume.

## Data Availability

After deidentification, the individual participant data that underlie the results reported in this article (text, tables, figures and appendices), as well as study protocol, will be available for researchers who provide a methodologically sound proposal. Proposals may be submitted after 9 months and up to 36 months following article publication.
